# Prevalence and demographic correlates of online grocery shopping: results from a nationally representative survey during the COVID-19 pandemic

**DOI:** 10.1017/S1368980022001756

**Published:** 2022-11

**Authors:** Emily W Duffy, Amy Lo, Marissa G Hall, Lindsey Smith Taillie, Shu Wen Ng

**Affiliations:** 1 Department of Nutrition, University of North Carolina, Chapel Hill Gillings School of Global Public Health, Carolina Population Center, 123 W Franklin St., Chapel Hill, NC 27516, USA; 2 Department of Health Behavior, University of North Carolina, Chapel Hill Gillings School of Global Public Health, Carolina Population Center, UNC Lineberger Comprehensive Cancer Center, School of Medicine, Chapel Hill, NC, USA

**Keywords:** Food retail, Nutrition, Diet, Food, Beverages

## Abstract

**Objective:**

To estimate the prevalence of online grocery shopping in a nationally representative sample and describe demographic correlates with online grocery shopping.

**Design:**

The Nielsen COVID-19 Shopper Behavior Survey was administered to a subset of Nielsen National Consumer Panel participants in July 2020. We used survey weighted-multivariable logistic regression to examine demographic correlates of having ever online grocery shopped.

**Setting:**

Online survey.

**Participants:**

18 598 Nielsen National Consumer Panel participants in the USA.

**Results:**

Thirty-nine percent of respondents had purchased groceries online, and among prior purchasers, 89 % indicated that they would continue to online grocery shop in the next month. Canned/packaged foods were the most shopped for grocery category online, followed by beverages, fresh foods and lastly frozen foods. In adjusted analyses, younger respondents (39 years or less) were more likely (47 %) to have ever shopped for groceries online than older age groups (40–54 years, 55–64 years and 65+ years) (29 %, 22 % and 23 %, respectively, all *P* < 0·001). Those with greater than a college degree were more likely to have ever grocery shopped online (45 %) than respondents with some college education (39 %) and with a high school education or less (32 %) (both *P* < 0·001). Having children, having a higher income and experiencing food insecurity, particularly among higher income food-insecure households, were also associated with a higher probability of prior online grocery shopping.

**Conclusions:**

The COVID-19 pandemic accelerated the transition to online grocery shopping. Future research should explore the nutrition implications of online grocery shopping.

The grocery retail sector is critical for public health intervention given 68 % of all foods consumed are consumed at home^(1)^. Online grocery shopping is becoming more popular as online shopping more generally has become the norm^(2,3)^. Online grocery shopping was already the fastest growing sector of online retail^(4)^, but the COVID-19 pandemic accelerated its use^(5,6)^. There are potential public health benefits of online grocery shopping such as improving geographical food access, reducing pester power from children accompanying parents shopping and reducing impulse purchases of unhealthy foods often caused by the physical food environment^(7–9)^. However, there is also potential for public health harms such as targeted junk food marketing, impulse purchases due to pop-up or banner advertisements and hesitancy to purchase fresh foods online^(8,10)^.

The health implications of online grocery shopping remain unclear as there is little research on online grocery shopping and nutrition in the USA. Only one study in the USA has compared online to in-store purchases within shoppers and found online purchases were associated with less candy, frozen desserts and grain-based desserts^(7)^. Similarly, few studies have examined the demographics of self-reported online grocery shoppers in the USA^(2,6,11)^ and have generally found that online grocery shoppers were more likely to have children, be younger, be female and have higher incomes. However, these studies used data from 2017 and have limited generalisability due to demographically homogenous or small samples of online shoppers.

Given the rapid shift in grocery shopping behaviours created by COVID-19, more representative and recent estimates of the prevalence of online grocery shopping and demographic characteristics associated with online grocery shopping in the USA are needed. Prior reviews have discussed the theoretical pitfalls and benefits of online grocery shopping, particularly in the context of a pandemic^(8,9)^; however, to our knowledge, no US studies have documented the prevalence of or demographic correlates of online grocery shopping using a nationally representative sample. This information can inform how to target policies, interventions and retailer initiatives designed to support groups at risk of diet-related disease in making healthy choices online.

The objectives of this research brief were to (1) estimate the prevalence of prior online grocery shopping in a nationally representative sample using data collected during the COVID-19 pandemic in July 2020; (2) report the proportion of households that planned to continue to online grocery shop in the future and (3) describe demographic correlates of prior (ever) online grocery shopping.

## Methods

### Participants and recruitment

In July 2020, Nielsen administered the Coronavirus Summer Survey to a subset of its National Consumer Panel participants. This online survey was one of two cross-sectional national surveys Nielsen administered (in April 2020 and July 2020) to assess consumer behaviour during the COVID-19 pandemic. A total of 18 598 households started the Summer Survey and 18 561 competed the survey. Nielsen asked each household’s primary shopper to complete the survey, which we assume is also the primary food shopper. Nielsen developed sampling weights for this survey that we have applied in all analyses, so results are nationally representative. This study was reviewed by UNC Chapel Hill’s Institutional Review Board and deemed non-human subjects research.

### Demographic measures

Respondent education, age and income were assessed via self-report. We used reported household size to recalculate continuous household income as a proportion of the 2020 Federal Poverty Level (FPL) index and created three categories of FPL index based on eligibility for federal nutrition assistance and other income support programmes in the USA. Race was measured by Nielsen using racial self-classification^(12)^ based on four closed-ended options: White, Black, Asian and Other Race. We do not know which other race categories are included in the Other Race category; however, it is important to note this group is very heterogeneous and does not necessarily represent a set of shared characteristics or experiences. Hispanic ethnicity was measured as a dichotomous variable. We recategorised race and Hispanic ethnicity into four groups based on small cell sizes of the Asian and other race groups: Non-Hispanic (NH) White, NH Black, NH Other Race (includes NH Asian) and Hispanic. Race and Hispanic ethnicity in our analyses are not indicators of biological differences but are representations of the sociopolitical processes that differentially impact individuals and their food-related behaviours^(13)^. Household food insecurity was assessed by Nielsen using a modified version of United States Department of Agriculture’s Household Food Security Survey Module: Six-Item Short Form. The recall period for these items was either over the last month or since the start of the pandemic (see online Supplemental File 1)^(14)^. For example, one of the items was ‘For the following statements, please tell us whether the statement was often true, sometimes true, or never true for you/your household *in the past month*. The food that I/my household bought just didn’t last, and I/my household didn’t have money to get more’.

### Online grocery shopping measures

Self-reported prior (ever) online grocery shopping behaviour was assessed using one item that was used to ask about four food or beverage products (e.g. canned/packaged foods and fresh foods) and six non-food products (e.g. paper products and household items): ‘Which statement best describes how you have shopped or would consider shopping for each of the following types of products? By “purchase online” we mean ordering any item through a website or mobile application for delivery or pick-up. Select one answer for each type of product: (1) Have purchased online and will buy online more in the future; (2) Have purchased online and will buy online the same amount in the future; (3) Have purchased online and will buy online less in the future; (4) Have not purchased online but might consider purchasing online in the future; and (5) Do not ever plan to purchase this product online’ (see online Supplemental File 1). If the respondent selected 1, 2 or 3 for any of the food or beverage categories, they were coded as an online grocery shopper (i.e. binary ever/never variable). Future online grocery shopping was similarly assessed using one item used to ask about a variety of products: ‘How much of each type of product do you expect to purchase online in the next month?’ This item was only asked about products that participants stated they had previously purchased online. If the respondent stated they intended to purchase any amount online of any food or beverage categories, they were coded as a future online grocery shopper.

### Data analysis

Our analytic sample included 18 124 households (of the 18 598 that started the survey) because we used complete cases. We ran survey-weighted descriptive statistics for items about prior (ever) online grocery shopping overall and by food category (e.g. beverages, frozen food) and for the item about future online grocery shopping. To examine demographic correlates of prior online grocery shopping, we used survey-weighted multivariable logistic regression. We used Stata’s margins command to compare adjusted predicted probabilities of prior online grocery shopping. Models were adjusted for respondent sex, age, education, the 2020 FPL index, race/ethnicity, children, food security and change in financial situation. In an exploratory analysis, we used an interaction term between FPL index and food security to explore moderation of the relationship between food security and prior online shopping by self-reported income. The level of statistical significance was determined by the Holm–Bonferroni method for adjusting for multiple comparisons, which achieves the same goal as the Bonferroni method (keeping the probability of one or more false discoveries below a certain level) but is less ‘costly’ in terms of statistical power^(15)^. We applied this method to control the familywise type 1 error rate at a level of 0·05 within each household characteristic. We conducted all analyses in Stata 16.

## Results

### Sample demographics

Table 1 shows the weighted and unweighted demographic characteristics of the sample. Of the 18 124 respondents with complete data, most identified as female (75 %) and about half of the sample was 55 years or older (46 %). Most respondents (69 %) did not have children in their household. Most households were food secure (79 %) and about three-fourths were middle (40 %) or high income (34 %). About one-third of respondents had a college education or greater (37 %). Two-thirds of respondents were NH White (67 %), followed by Hispanic (14 %), NH Black (12 %) and NH Other Race individuals (7 %). About one-fourth of respondents (27 %) indicated that their financial situation was worse than at the start of the pandemic, and 30 % reported spending their stimulus check on food or household items.

**Table 1:** Sample characteristics of respondents (*n* 18 124*)

Characteristics	Unweighted prevalence (%)	Weighted prevalence (%)
Sex		
Female	80	75
Male	20	25
Age		
39 years or younger	16	25
40–54 years	26	29
55–64 years	27	22
65 years or older	32	24
Education		
Greater than college	16	13
College graduate	33	24
Some college	30	31
High school or less	22	31
FPL index		
>400 % FPL	33	34
185 %–400 % FPL	44	40
<185 % FPL	23	27
Race/ethnicity		
NH White	77	67
NH Black	9	12
NH other race	7	7
Hispanic	7	14
Presence of children (<18 years)		
No	80	69
Yes	20	31
Food insecure		
No	82	79
Yes	18	21
Change in financial situation		
Same or better than at the start of the pandemic	73	73
Somewhat or much worse than at the start of the pandemic	27	27
Used stimulus check on food and household items		
No	71	70
Yes	29	30

FPL, federal poverty level; NH, non-Hispanic.

* Education data missing from 437 households.

Authors’ calculations based in part on data reported by NielsenIQ in its COVID-19 Shopper Behavior Surveys, NielsenIQ, 2020. The conclusions drawn from the NielsenIQ data are those of UNC and do not reflect the views of NielsenIQ. NielsenIQ is not responsible for and had no role in, and was not involved in, analysing and preparing the results reported herein.

### Self-reported online grocery shopping behaviours

**Fig. 1:**
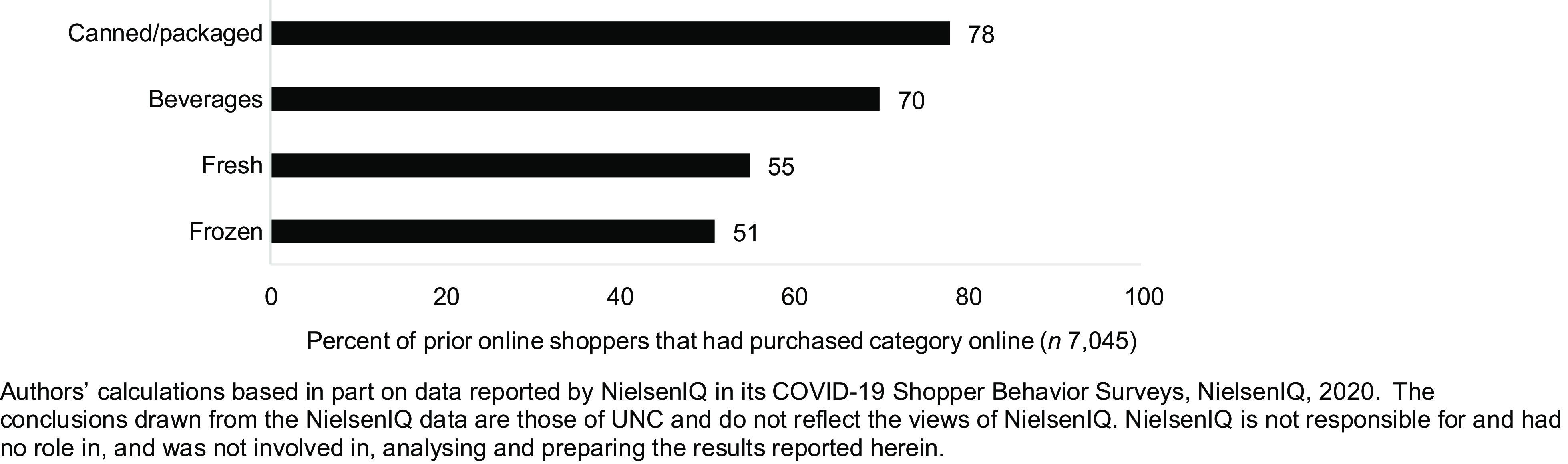
Prevalence of online grocery shopping by food category among online grocery shoppers (*n* 7045)

Thirty-nine percent of respondents had ever purchased groceries online, and among those who reported prior purchases (*n* 7045), 89 % of prior online shoppers indicated that they would continue online grocery shopping in the next month. Among those who had grocery shopped online (*n* 7045), canned/packaged foods were the most shopped for category online (78 % reported prior purchases), followed by beverages (70 %), fresh foods (55 %) and lastly, frozen foods (51 %) (Fig. 1). The full range of responses to this item can be found in Supplemental Fig. 1.

### Demographic correlates of online grocery shopping

In adjusted analyses, females were more likely (40 %) to have online grocery shopped than males (34 %) (*P* < 0·001) (Table 2). Respondents 39 years or younger were more likely (47 %) to have online grocery shopped than those 40–54 years (41 %), 55–64 years (36 %) and 65 years or older (31 %) (all *P* < 0·001). Compared with respondents with greater than a college degree (45 %), those with some college education (39 %) and a high school education or less (32 %) were less likely to have grocery shopped online (both *P* < 0·001). The presence of children was also associated with a higher likelihood of online grocery shopping (41 % compared with 38 % in households with no children, *P* = 0·03). Food-insecure households were more likely to have grocery shopped online than food-secure households. This relationship was strongest among higher income households (56 % of higher income food-insecure households and 39 % of higher income food-secure households, *P* < 0·001) than for middle (47 % of food-insecure and 38 % of food-secure households, *P* < 0·001) and low-income households (37 % of food-insecure and 34 % of food-secure households *P* = 0·08) (Table 2). There were no statistically significant differences in the predicted probability of online grocery shopping by race/ethnicity. There were also no differences by change in financial situation.

**Table 2:** Correlates of reported prior online grocery shopping behaviour (*n* 18 124)

Characteristics	Unadjusted prevalence (%)	Multivariable adjusted predicted probabilities	95 % CI	*P*
Sex				
Male (ref)	33	34	32–36	
Female	41	40*	39–41	<0·001
Age				
39 years or younger (ref)	50	47	45–49	
40–54 years	43	41*	39–43	<0·001
55–64 years	34	36*	34–38	<0·001
65 years or older	28	31*	29–32	<0·001
Education				
Greater than college (ref)	46	45	43–48	
College graduate	45	44	42–46	0·38
Some college	39	39*	37–41	<0·001
HS or less	31	32*	30–34	<0·001
FPL index and food security				
>400 % FPL and food secure (ref)	39	39	37–41	
>400 % FPL and food insecure	60	56*	50–62	<0·001
185 %–400 % FPL and food secure (ref)	38	38*	37–40	
185 %–400 % FPL and food insecure	49	47*	44–51	<0·001
<185 % FPL and food secure (ref)	33	34	32–36	
<185 % FPL and food insecure	40	37	35–40	0·08
Race/ethnicity				
NH White (ref)	38	39	38–40	
NH Black	37	36	33–39	0·05
NH other race	43	39	36–42	0·96
Hispanic	45	41	38–44	0·35
Presence of children (<18 years)				
No (ref)	36	38	37–39	
Yes	46	41*	39–43	0·03
Change in financial situation				
Same or better than before pandemic (ref)	38	38	37–40	
Somewhat or much worse than before pandemic	43	40	38–42	0·13

FPL, federal poverty level; NH, non-Hispanic.

* Statistically significantly different after using the Holm method to control the familywise error rate at level 0.05 within each household characteristic. Confidence intervals not adjusted for multiple comparisons.

Authors’ calculations based in part on data reported by NielsenIQ in its COVID-19 Shopper Behavior Surveys, NielsenIQ, 2020. The conclusions drawn from the NielsenIQ data are those of UNC and do not reflect the views of NielsenIQ. NielsenIQ is not responsible for and had no role in and was not involved in analysing and preparing the results reported herein.

## Discussion

In a nationally representative survey conducted in July 2020, 39 % of households in the US had ever shopped online for groceries. This prevalence estimate is likely higher than what it would have been before the COVID-19 pandemic because we know some people were grocery shopping online for the first time during the pandemic^(16)^. Packaged/canned foods and beverages were more commonly purchased online than fresh or frozen foods, potentially due to their ease of transport and longer shelf life. Also, given the timing of the survey and governmental recommendations to have 2 weeks supply of food on hand at the start of the pandemic, it is possible US shoppers had stocked up on canned/packaged foods to prepare for the pandemic. This has important potential public health implications as other studies have highlighted hesitancy to purchase fresh, perishable items online^(8)^. Almost all respondents who had online grocery shopped planned to continue online grocery shopping in the next month. Future studies need to examine whether online grocery shopping behaviours persist after the impact of the pandemic on people’s day-to-day behaviours subsides. Like prior studies^(11,17)^, we found women were more likely than men to report online grocery shopping as were households with children compared with households without children. We also found younger, higher income, and higher educated respondents were more likely to report prior online grocery shopping. There were no statistically significant differences in prior online shopping by race/ethnicity; however, this study did not assess differences in frequency or amount of online grocery shopping which should be explored in further studies. Interestingly, in our sample, food-insecure households were more likely to have online grocery shopped, and this pattern was particularly pronounced among higher income households reporting food insecurity. It is possible that these households had online grocery shopped prior to the pandemic and then experienced job loss or furlough because of the pandemic leading to food insecurity. For example, 59 % of high-income households reporting food insecurity reported their financial situation was worse than before the pandemic compared with 27 % of the total sample. This relationship could simply be a matter of the timing of the measures. It is also possible that reporting food insecurity is associated with a higher likelihood of living in an area with limited food retailers so food-insecure households may use online grocery shopping to cope with limited food access. Also, the FPL index is not adjusted for cost of living, so these households may have high incomes according to the FPL index but live in an area with a high cost of living. Similarly, although the difference was NS, households that self-reported being somewhat worse off financially since the start of the pandemic were more likely to have ever online grocery shopped than households that reported being the same or better off financially. Ultimately, this study cannot determine why these associations were observed, and these relationships should be explored in future studies using more appropriate methods.

While there is an emerging body of literature documenting online grocery shopping behaviours and shifts in self-reported food-related behaviours (e.g. snacking) cooking, fruit and vegetable consumption) during the pandemic^(5,18–20)^, only one study has reported an estimate of the prevalence of online grocery shopping in the USA during COVID-19^(5)^. This study was conducted earlier in the pandemic (March – April of 2020) and reported lower prevalence (10–15 %) of online grocery shopping^(5)^. Our findings are consistent with the few studies that have examined demographic correlates of online grocery shopping in the USA (female, higher income, children in household more likely to grocery shop online)^(2,11)^. In addition to understanding the demographic characteristics of online grocery shoppers, future studies should also compare the quality of food purchases online and in store. Additionally, future studies should explore if similar relationships between online grocery shopping and demographic characteristics exist in non-US contexts.

With the expansion of online grocery shopping, there is interest in public health policies and interventions to facilitate healthier food choices online. From the available evidence, it seems that higher income, higher educated, women with children may be making the transition from in store to online grocery shopping more quickly than comparator groups^(11)^. However, lower income and food-insecure shoppers are also transitioning to online grocery shopping, as 36 % of the lower income participants in our sample had grocery shopped online. Additionally, during the COVID-19 pandemic the United States Department of Agriculture started allowing Supplemental Nutrition Assistance Program benefits to be used for online grocery shopping and subsequently Supplemental Nutrition Assistance Program benefits redeemed online grew from $2·9 billion in February 2020 to $196·3 billion in September 2020. This transition paired with United States Department of Agriculture likely approving the Special Supplemental Nutrition Program for Women, Infants and Children use for online grocery shopping in the near future accelerates the need to identify strategies that promote healthy choices online^(21)^.

To promote equitable access to online grocery shopping, the public health community should proactively be considering solutions to barriers to low-income and food-insecure groups grocery shopping online identified in prior research such as processing fees, lack of control over food selection, minimum order requirements and inconvenient delivery times^(22–26)^. Additionally, as groups traditionally more burdened by diet-related chronic diseases begin grocery shopping online, policies must be in place to protect shoppers from targeted junk food marketing^(10)^ such as requiring Supplemental Nutrition Assistance Program retailers to highlight healthy options and for the Federal Trade Commission to study how race, income and address are used to inform promotions in online grocery stores^(27)^. Finally, policies targeting other determinants of food intake such as pricing (e.g. fruit and vegetable incentives or junk food taxes) and physical access to healthy food will need to be considered. The retail sector is of critical importance to diet-related disease prevention as the vast majority of food intake occurs at home and even more so since the start of the pandemic.

While this study provides a nationally representative estimate of the prevalence of online grocery shopping and characterisation of demographic groups that may be more likely to grocery shop online, there are several important limitations. Our primary outcome measure is a relatively crude measure of having ever purchased groceries online and does not provide information on the frequency, timeframe or preferred retailer(s) of online grocery shopping. Additionally, as our team was not involved in the design or administration of this Nielsen survey, it is unknown if the measures used in the Nielsen NCP summer survey were validated or cognitively tested. Finally, the national sample could be considered both a strength and limitation, as the COVID-19 pandemic likely affected grocery shopping behaviours of communities in the USA in differential ways. Future studies should explore geographic variability in food shopping behaviours during the pandemic.

In conclusion, among a nationally representative sample in July 2020, 39 % of respondents had ever shopped for groceries online and most of those respondents planned to grocery shop online in the future. In our sample, characteristics such as being female, having a child, being higher educated, higher income and younger as well as being food insecure were associated with a higher likelihood of having grocery shopped online. As online grocery retail continues to grow, the public health community should be considering strategies that prevent online grocery shopping from exacerbating existing nutrition-related disparities.

## Supplementary material

For supplementary material accompanying this paper visit https://doi.org/10.1017/S1368980022001756. click here to view supplementary material
