# Supplementary material for: Prevalence and demographic correlates of online grocery shopping: results from a nationally representative survey during the COVID-19 pandemic
Source: Public Health Nutr. 2022 Aug 19;25(11):3079–85. doi: 10.1017/S1368980022001756 (PMC9464506; doi:10.1017/S1368980022001756)
Supplement: Supplementary file 1 [file S1368980022001756sup.zip › S1368980022001756sup001.docx]

**Supplemental File 1.** Survey Item Wording and Coding from Nielsen COVID-19 Shopper Behavior Survey

**Self-Reported Change in Financial Situation**

| Since the start of COVID-19 my household financial security is... | () Much better  () Somewhat better  () About the same  () Somewhat worse  () Much worse |
| --- | --- |

This item was used to create a binary variable of financial security being the same, somewhat better or much better and financial security being worse or much worse.

**Food Insecurity (modified using 5 items from USDA’s 6-item scale)**

We would like to know more about the food situation in your household during the ***past month***. For the following statements, please tell us whether the statement was often true, sometimes true, or never true for you/your household ***in the*** ***past month***.

| 1. The food that I/my household bought just didn’t last, and I/my household didn’t have money to get more. 2. I/my household couldn’t afford to eat balanced meals. | () Often true  () Sometimes true  () Never true  () Don’t know / Prefer not to answer |
| --- | --- |

For the following statements, please tell us *how often* each was true for you/your household ***since the coronavirus outbreak***.

| 3. Since the coronavirus outbreak, I/others in my household cut the size of our meals or skipped meals because there wasn't enough money for food. 4. Since the coronavirus outbreak, I/others in my household ate less than I felt we should because there wasn't enough money for food. 5. Since the coronavirus outbreak, I/others in my household were hungry but didn't eat because there wasn't enough money for food. | () Once or twice  () About once a week  () Daily  () Never  () Don’t know / Prefer not to answer |
| --- | --- |

These five items were used to construct a score for food insecurity. If the respondent selected sometimes true or often true for the first two items (food did not last, could not afford to buy balanced meals), they were given a score of one for those items. If the respondent selected daily, once a week, or once or twice for the following three items (cut size/skipped meals, ate less because there wasn’t enough money for food, hungry), they were given a score of one for those items. The scores of 0/1 for those five items were then summed for each respondent and if they had a score greater than or equal to two, they were deemed food insecure and if they had a score less than two, they were deemed food secure. This scoring is consistent with how the USDA scores their 6-item food security measure.

**Prior Online Grocery Shopping (ever)**

Which statement best describes how you have shopped or would *consider* shopping for each of the following types of products? By “**purchase online**” we mean ordering an item through a website or mobile application for delivery or pick-up. Select one answer for each type of product.

| Canned / Packaged foods (e.g., shelf stable foods)  Beauty / Grooming Products (e.g., hair, skin and body care)  Baby products (e.g., diapers, wipes, formula, etc.)  Pet care (e.g., pet food, treats, etc.)  Household items (e.g., cleaning products or tools, air fresheners, etc.)  Paper Products (e.g., bathroom tissue, facial tissue, paper towels, etc.)  Healthcare items (e.g., non-prescription medicine, vitamins, minerals, etc.)  Fresh foods (e.g., dairy, produce, meats, prepared foods, etc.)  Frozen foods (e.g., frozen meals, vegetables, desserts, etc.)  Beverages (e.g., milk, juice, tea, coffee, water, soda, etc.) | (1) Have purchased online and will buy online **more** in the future  (2) Have purchased online and will buy online **the same amount** in the future  (3) Have purchased online and will buy online **less** in the future  (4) Have not purchased online but might consider purchasing online in the future  (5) Do not ever plan to purchase this product online |
| --- | --- |

If the respondent selected one, two, or three for any of the food or beverage categories, they were coded as an online grocery shopper (i.e., binary ever/never variable).

**Future Online Grocery Shopping (in the next month)**

How much of each type of product do you expect to purchase online ***in the next month***? By “**purchase online**” we mean ordering an item through a website or mobile application for delivery or pick-up. Select one answer for each type of product.

| Canned / Packaged foods (e.g., shelf stable foods)  Beauty / Grooming Products (e.g., hair, skin and body care)  Baby products (e.g., diapers, wipes, formula, etc.)  Pet care (e.g., pet food, treats, etc.)  Household items (e.g., cleaning products or tools, air fresheners, etc.)  Paper Products (e.g., bathroom tissue, facial tissue, paper towels, etc.)  Healthcare items (e.g., non-prescription medicine, vitamins, minerals, etc.)  Fresh foods (e.g., dairy, produce, meats, prepared foods, etc.)  Frozen foods (e.g., frozen meals, vegetables, desserts, etc.)  Beverages (e.g., milk, juice, tea, coffee, water, soda, etc.) | () Will buy all of it online  () Will buy most, but not all, online  () Will buy about half online and half in a store  () Will buy some online, but mostly from a store () Will stop buying any online |
| --- | --- |

This item was only asked about products that participants stated they had previously purchased online. If the respondent stated they intended to purchase any amount online of any food or beverage categories, they were coded as a future online grocery shopper.
