# Supplementary material for: Prevalence and demographic correlates of online grocery shopping: results from a nationally representative survey during the COVID-19 pandemic
Source: Public Health Nutr. 2022 Aug 19;25(11):3079–85. doi: 10.1017/S1368980022001756 (PMC9464506; doi:10.1017/S1368980022001756)
Supplement: Supplementary file 1 [file S1368980022001756sup.zip › S1368980022001756sup003.docx]

**Supplemental Figure 1.** Prevalence of online grocery shopping behaviors by food category among the total sample (n=18,561)

Survey weighted percent of sample

|  | Never plan on buying online | Have not but may in future | Have and will buy less in future | Have and will buy same in future | Have and will buy more in future |
| --- | --- | --- | --- | --- | --- |
| Canned | 44 | 25 | 5 | 17 | 9 |
| Fresh | 62 | 16 | 4 | 11 | 6 |
| Frozen | 63 | 17 | 3 | 11 | 6 |
| Beverages | 54 | 19 | 4 | 16 | 8 |

Authors’ calculations based in part on data reported by NielsenIQ in its COVID-19 Shopper Behavior Surveys, NielsenIQ, 2020.  The conclusions drawn from the NielsenIQ data are those of UNC and do not reflect the views of NielsenIQ. NielsenIQ is not responsible for and had no role in, and was not involved in, analyzing and preparing the results reported herein.
